# Gunshot Wounds of Peripheral Nerves

**Published:** 1917-07

**Authors:** J. Michell Clarke

**Affiliations:** Lieut.-Col., R.A.M.C.(T), Professor of Medicine, University of Bristol


### GUNSHOT WOUNDS OF PERIPHERAL NERVES.

BY

J. MICHELL CLARKE, M.D., F.R.C.P.,

*Lieut.-Col., R.A.M.C.(T),*

*Professor of Medicine, University of Bristol.*

---

INJURIES of peripheral nerves occur in wounds due to projectiles of high velocity with great frequency, and their successful treatment is of the highest importance in the prevention of permanent disabilities of the most serious kind. It may be said at once that we are not yet in a position to make positive statements as to what the ultimate results of nerve injuries and the operations undertaken for their relief will be.

The nerves most frequently affected are the musculo-spiral, median and ulnar in the arm, the sciatic, and especially its external popliteal branch, in the leg.

Injuries to the brachial plexus are also common.

A nerve may be (1) completely divided—when this is so there is rarely a clean cut through it, but more often destruction or obliteration of it for a considerable distance, from one to several inches; or (2) partially divided, or (3) injured by compression in scar-tissue, by callus, by fragments of bone, by a lodged bullet, or portions of clothing, or by a traumatic aneurysm, by bruising or concussion.

It is important to remember that perineural scars and adhesions, though conspicuous at operation, may not be responsible for blocking the passage of impulses, but that this may be due to concomitant intra-neural hemorrhage, fibrosis (neuroma), or molecular changes of concussion. Trotter and Davis have shown the intolerance of the ordinarily isolated nerve-tissue to the contact of connective tissue, and that the active out-growth of nerve fibrils of a divided nerve is a response to the contact of non-nervous tissue, which on its part forms a tough, fibrous capsule around them, and prevents their further extension; this may lead to irritation of the nerve and hyper-sensitiveness.

The extensive experiences of the war should give more exact knowledge as to the course in the nerves of the individual nerve bundles to different muscles, information which is much needed for more exact plastic surgery.

The location of the injury to a nerve rests on anatomical data, and on the course of the projectile, aided by X-rays as to the presence of foreign bodies. In multiple wounds the diagnosis of the one causing the lesion is made from anatomical considerations. The extent of damage to the nerve is estimated by investigation of motor and sensory paralysis, muscular wasting, pains and hyperæsthesia, trophic lesions, and electrical reactions, and all of these symptoms which are present should be compared, and the diagnosis based upon the full results. There is no short cut to diagnosis. Many consider that sensory changes give more important information both as to extent and progress of the lesion than motor paralysis or electrical reactions.

It has been claimed that in incomplete division of a nerve the area of loss to protopathic is greater than to epicritic sensation, and that the borders of the respective areas approach and recede from or cross each other; but this is not yet established.

It is certain that the distinction between complete anatomical division of a nerve and physiological blocking of it by involvement in scar tissue cannot always be made, for full sensory and motor loss with R.D. may be present in the latter.

Pains and hyperæsthesia mean partial injury of a nerve; when these gradually disappear and give place to analgesia and tactile anæsthesia compression of the nerve by scar tissue, callus, etc., is probably taking place.

Hysterical anæsthesia of the sleeve, glove or stocking type may be present as a complication of peripheral nerve-injuries, generally in partial ones. In gunshot wounds, however, especially of plexuses, the symptoms are often very complicated, and the sensory loss may not at first correspond with the known anatomical distribution of the nerves, and may resemble hysterical anæsthesia, but after a few weeks partially clears up leaving a residual loss in recognised areas of nerve-supply.

The severe forms of trophic disturbance, such as deep and intractable ulcers, usually occur in complete division of a nerve, the milder forms in partial lesions.

A lesion partial for a whole nerve may, however, be complete for one of its bundles of fibres ; this is not infrequent in the sciatic, where a wound of the nerve as high as the gluteal region may completely divide the external or internal popliteal fibres, most often the former, and leave the other little or not at all affected.

Much discussion has taken place on electrical reactions of damaged nerves. Electrical reactions give no information as to what is going on in the nerve at the seat of injury. Their most important function is to decide whether there are any excitable fibres peripheral to the injury. When all have been lost, voluntary power usually returns before the muscles respond to Faradism or the condenser. This fact, that electrical response does not go *pari passu* with voluntary power, is the chief reason why electrical reactions give such limited information as to extent of injury to nerve.

The Lewis Jones Condenser is now largely used for taking electrical reactions. Its great advantages are that it is reliable, gives sufficient information, is painless, and saves time. On the other hand, it has been shown that the condenser discharges vary with the resistance and other factors, and that the patient's resistance is not constant, as for practical purposes it was at first assumed that it would be, and therefore caution is necessary in estimating the progress of a lesion by condenser reactions, for which it has been especially advocated. Further, the ready diffusion of the current renders its use difficult for small muscles. The advantages of the condenser, however, greatly outweigh its defects, and although some think that more information is to be gained from the older methods, it seems likely to come into general use.

As to treatment. Operations on cases in this country will be for secondary suture. Before operation can be done, (1) the wound must be soundly healed; (2) there must be a sufficient interval after the injury before exact diagnosis is possible, partly because characteristic signs of division of a nerve take time to develope, and partly because in most cases of gunshot wounds of nerves the extent of paralysis is at first more extensive than remains permanently.

Especially in wounds of a plexus, the immediate loss of sensation and motion may affect the whole limb, and after a time partially clear up, leaving a residual paralysis.

Whilst waiting, preliminary treatment must be carried out. This consists in wrapping the limb up warmly to protect it from cold and injury; in placing it on a suitable splint to keep the paralysed muscles relaxed—a point of the greatest importance; daily gentle massage and movements, during which the paralysed muscles must be kept relaxed; and electrical treatment by the passage of a constant current, or condenser discharge, rhythmically interrupted by a metronome, and just strong enough to cause a contraction of the muscle.

It is desirable before operation to give a prophylactic dose of tetanus antitoxin, as tetanus may develop after operation undertaken some months after the original injury.

Operation is advisable (and, *ceteris paribus*, the earlier performed the better the results) (1) in complete division of a nerve ; (2) in incomplete lesions (*a*) where the condition is stationary with a definite residue of paralysis after three to four months, (*b*) where improvement has begun but stopped abruptly, with signs of compression of the nerve by scar-tissue, callus, etc., (*c*) where there is great pain, not relieved after some weeks' careful treatment, the nerve should always be explored (pieces of bone, foreign bodies, or shreds of clothing may be found in the nerve) ; and (3) in cases of old standing with intractable trophic lesions, for even if no good to the paralysis results, the trophic lesions quickly heal.

In the majority of cases there is little difficulty in deciding whether operation is necessary or not. Even slight return of voluntary power negatives operation. In doubtful cases nothing is lost and often much gained by an exploratory operation, though occasionally the nerve may be found to be apparently normal in cases where the paralysis was complete. This is especially true of the musculo-spiral nerve.

In plexus lesions where several trunks have been divided, often at different levels with much perineural scar-tissue, accurate identification of trunks and end to end suture is most difficult, and if found impossible, it is probably better to leave things alone than to do indiscriminate nerve crossing. Trotter advises early operation in cauda equina lesions in every case where there is the least suspicion of an element of pressure.

The post-operative treatment, and that of cases of partial division of nerves is the same as given above in preliminary treatment, and should be continued daily until there is a return of voluntary impulses, which may take weeks or years. In the lower extremity a walking instrument must also be provided for dropped feet. After end to end suture of a nerve electrical treatment should not be begun for some little time, and then with great care.

In all cases, especially in the early stages, care must be taken to avoid fatigue of the muscles by electrical treatment; the balance of evidence is that contractions due to electrical stimulation are beneficial in the stage of regeneration, but have no effect on muscular wasting. In later stages there may be irreparable paralysis and joint or bone lesions so severe as to neutralise the effect of recovery of paralysed muscles ; for such cases operations for tendon transplantation or arthrodesis may be advisable.

As to results. Cases of nerve concussion should recover within four to six weeks ; partial division of a nerve should go to complete recovery in several months (? two to six).

As to cases of complete division with secondary suture, it is not yet time to speak as to the results. Meanwhile, some statements of recent experiences seem to show a much earlier return of sensation and motion than was before held possible. The greater the distance from the periphery the site of the lesion the longer is the period before recovery.

---

### Operative Procedures and Findings, by Capt. Rendle Short, M.S., F.R.C.S.

1. The nerve may appear to be normal (*vide supra*).

2. Complete division of the nerve, with a bulbous swelling at the end of the upper segment, succeeded by a mass of scar-tissue of variable thickness, and adherent to the surrounding tissues. The treatment adopted is to free the two ends of the nerve, cut off the upper segment through the upper end of or above the bulbous swelling, so as to expose visible nerve-bundles with no excess of interstitial fibrous tissue, and suture with catgut. Many English surgeons wrap the junction in Cargile membrane; others sometimes use a segment of saphena vein, but often nothing is used.

It frequently happens that the ends cannot be brought together because of a gap of one to two inches. This is bridged by two or three plies of internal cutaneous nerve from the upper arm, but some surgeons use catgut strands, and others employ nerve-anastomosis.

3. There may be a bulbous swelling on the nerve, adherent to the track of the bullet, and it may be doubtful whether the nerve-fibres pass through it or are interrupted. In such a case we stimulate the nerve electrically above and below the bulb. If, in the case of a small or medium-sized nerve (not the sciatic), any muscles supplied by it contract, the scar is freed and wrapped in a segment of saphena vein. If there is no response, we excise the scar and suture.

4. A large nerve, such as the sciatic, may show symptoms of a partial division, and at operation part of the nerve shows a scar. The best procedure is to take out a quadrilateral, including the scar, and, by splitting the nerve-trunk up and down, to bring the two ends together, leaving the intact portion of the nerve undisturbed.

5. The nerve may be intact, but pressed upon by a bullet or by shattered bone. The treatment is to remove the cause of pressure, but although this relieves pain at the time, it is apt to return during the process of healing, and prove very intractable.

---

### REFERENCES.

*Nerve Injuries and their Treatment*, Purves Stewart & Evans, London, 1916.

"Discussion on Gunshot Wounds of the Peripheral Nerves," *Medical Society's Trans.*, 1916, xxxix. 27.

Core, "Dissociation of Cutaneous Sensations in Injuries to Peripheral Nerves," *Lancet*, 1916, i. 716.

Stopford, "Peripheral Nerve Injuries," *ibid.*, 1916, ii. 718.

Langley, "Cause and Nature of Changes which occur in Muscles after Nerve Section," *ibid.*, 1916, ii. p. 6.

Langley, *ibid.*, 1916, ii. p. 161.

Hernaman Johnson, *ibid.*, 1916, ii. p. 120.

Hernaman Johnson, "Condenser Reactions," *Proc. Roy. Soc. Med.*, 1916, Surgical Section, p. 1.

Warrington and Nelson, "Musculo-Spiral Nerve Injuries," *Liverpool Med. Chir. Journ.*, 1916, lxix. p. 61.

Bernard Roth, "Gunshot Wounds of Peripheral Nerves," *Journ. R.A.M.C.*, 1915, i. p. 267.

Platt, "Treatment of Nerve Injuries of Warfare," *Clin. Journ.*, 1916, Aug. 9th., p. 285.

Adrian, "Electrical Reaction of Muscles before and after Nerve Injury," *Brain*, 1916, vol. xxxix., pt. i., p. 1.

Roberts, "Degeneration of Muscle following Nerve Injury," *Brain*, 1916, vol. xxxix., pt. iii., p. 297.

Cumberbatch, "Electrical Testing," *Proc. Roy. Soc. Med.*, 1914., Elect. Therap. Section, p. 38.

Purser, *Roy. Acad. Med. Ireland*, Section Surgery, 1916.

Tubby, "Nerve Concussion due to Bullet and Shell Wounds," *Brit. Med. Journ.*, 1915, i. p. 57.

